# A Walking Encyclopedia

**Published:** 1889-02

**Authors:** 


### A WALKING ENCYCLOPEDIA.

### MARVELOUS POWER OF MEMORY OF A SMALL NEGRO BOY.

Professor Oscar Moore gave a reception in parlor L, Astor House, a few days ago to representatives of the New York newspapers, which was one of the most remarkable ever seen in the city. Professor Moore was born near Waco, Tex., is colored, has been totally blind from birth, and is three years and four months old. What the sensitive plates in the phonograph are to sounds, Professor Moore's memory is to ideas. Facts, figures, dates, poetry, speeches on the tariff. whatever is thrown against that wonderful faculty of the diminutive professor lodges there and stays and the professor can go over all the vast stock that he has already accumulated in his brief life and produce any article he requires at an instant's notice.

He is a naturally developed child for his age, with a bright face and wide-open, handsome eyes, which have the queer appearance of looking inward instead of outward. The powers of his memory seem to be absolutely without limit. Whatever is repeated to him once or twice he never forgets. In response to questions, he told instantly the populations of all the nations of the earth and of all the cities. Statistics of weight and measurement involving sums of no matter how many figures, he rattled off before the question was hardly out of the questioner's mouth. He sang songs in German, Danish and English. He conversed in eleven different languages. He made a speech with laughable reproductions of all the exaggerated sounds, intonations and rising and falling inflections of the stump orator.

It is only about eight months since his memory began receiving its impressions under an instructor, and yet the child is literally a walking encyclopedia of useful information.

A gentleman from Austin, Tex., has him in charge, and, forseeing in him a possible fortune, induced the boy's parents to relinquish their authority for the next seventeen years. His father and mother are ignorant colored people, and were formerly slaves.

